# Exercise mediates ubiquitin signalling in human skeletal muscle

**DOI:** 10.1096/fba.2021-00142

**Published:** 2022-02-26

**Authors:** Samuel O. Lord, Yu‐Chiang Lai

**Affiliations:** ^1^ School of Sport, Exercise and Rehabilitation Sciences University of Birmingham Birmingham UK; ^2^ Institute of Metabolism and Systems Research University of Birmingham Birmingham UK; ^3^ Mitochondrial Profiling Centre University of Birmingham Birmingham UK; ^4^ Medical Research Council (MRC) Versus Arthritis Centre for Musculoskeletal Ageing Research University of Birmingham Birmingham UK

**Keywords:** mass spectrometry, NEDDylation, physical activity, ubiquitylation, ubiquitylome

## Abstract

Physical activity or regular exercise provides many beneficial effects towards human health, helping prevent and ameliorate metabolic diseases. However, certain molecular mechanisms that mediate these health benefits remain poorly understood. Parker et al. provided the first global analysis of exercise‐regulated ubiquitin signalling in human skeletal muscle, revealing post‐translational modification cross‐talk. As a result of their analysis, NEDDylation is thought to promote ubiquitin signalling for the removal of damaged proteins following exercise. The proteomic dataset generated from their study is invaluable for researchers in this field to validate new mechanistic hypotheses. To further reveal molecular mechanisms regulated by exercise, future research could employ more sensitive mass spectrometry‐based workflows that increase the detection of both ubiquitylated sites and peptides and subsequently identify more exercise‐regulated ubiquitin signalling pathways.

**Abbreviations**
cAMP: cyclic adenosine monophosphate
DDA: data‐dependent acquisition
DIA: data‐independent acquisition
diGLY: diglycine
K‐ε‐GG: ubiquitin remnant motif
PTMs: post‐translational modifications
TMT: tandem mass tag
TR‐TUBE: trypsin‐resistant TUBE
TUBE: tandem ubiquitin‐binding entity

Skeletal muscle is a dynamic tissue, crucial for whole‐body functioning due to its role in both locomotion and metabolism. To meet such multifunctional demands, skeletal muscle is highly adaptable and undergoes structural and metabolic remodelling. Exercise is a well‐established physiological stimulus for inducing skeletal muscle remodelling which maintains function. One of the key molecular drivers for promoting skeletal muscle remodelling is protein turnover. Protein turnover is essential for preventing the accumulation of damaged and misfolded proteins, which when impaired contributes to ageing and disease.^1^ Therefore, understanding the underlying mechanisms by which exercise induces protein turnover will be beneficial for developing new therapeutics to maintain skeletal muscle health.

Post‐translational modifications (PTMs), such as protein ubiquitylation and phosphorylation, are signalling messengers that regulate biological processes, such as protein turnover, in cells and tissue including skeletal muscle. However, research over the last few decades has primarily focused on protein phosphorylation. Accordingly, we now have considerable knowledge of protein kinase‐ and phosphatase‐dependent signalling pathways. In contrast, protein ubiquitylation remains poorly studied, especially in relation to exercise.

Ubiquitylation is most known for its role in protein degradation. The addition of ubiquitin to proteins by ubiquitin‐activating (E1), ‐conjugating (E2) and ‐ligating (E3) enzymes can signal for degradation via the proteasome. For this to occur, a specific ubiquitin modification must be formed. Ubiquitin can be bound to substrates as a monomer on a signal site (monoubiquitylation) or multiple sites (multi‐monoubiquitylation). Alternatively, ubiquitin can be polymerised to form a chain on the substrate (polyubiquitylation).^3^ Polyubiquitination can be classified into eight different chain types depending on the ubiquitin linkage site (K6, K11, K27, K29, K33, K48, K63 and M1). While certain ubiquitin chain types signal for non‐degradative processes, K48‐ and K11‐linked ubiquitin chains are known to signal for protein degradation via the proteasome.^4^

Due to its well‐known role in degrading proteins, ubiquitylation is thought to be important for removing damaged myofibrillar proteins after exercise. Previous studies have shown that exercise promotes muscle protein breakdown.^5^, ^6^ However, the underlying molecular mechanisms responsible are not fully understood. Support for ubiquitin‐mediated protein degradation comes from increased proteasome activity and subsequent decreases in K48‐linked ubiquitin chains following a single bout of high‐intense endurance exercise.^7^ These findings have meant that ubiquitin is often described as a ‘death marker protein’. Over the last few years, accumulating evidence has indicated that protein ubiquitylation is involved in many other biological regulations beyond protein degradation in skeletal muscle. For example, nonproteolytic ubiquitylation has shown to stabilise proteins required for sarcomeric integrity.^8^ Furthermore, while many ubiquitylated proteins are degraded by the proteasome, their removal can regulate non‐degradative signalling events such as myogenesis and insulin signalling.^9^ However, due to the lack of comprehensive research, the role of protein ubiquitylation in skeletal muscle following exercise remains incompletely understood.

A recent publication by Parker et al., is the first study to provide a global analysis of exercise‐regulated protein ubiquitylation in skeletal muscle.^10^ They found that a single bout of high‐intense endurance exercise alters 391 ubiquitylated peptides in 160 different proteins, revealing several ubiquitin‐mediated signalling pathways during exercise. Furthermore, they demonstrated that high‐intense exercise leads to dynamic changes in the abundance of each lysine‐linked polyubiquitylated chain. Overall, this study has provided a valuable resource of candidate proteins and ubiquitin modifications to assist future research into exercise‐regulated protein ubiquitylation, opening a multitude of avenues to be explored in greater depth.

In support of the notion that exercise promotes protein degradation through ubiquitylation, K48‐linked ubiquitin chains were rapidly restored to baseline levels following an immediate decrease during exercise.^10^ The authors proposed that NEDDylation is responsible for promoting ubiquitin‐mediated protein degradation following exercise. NEDDylation is a ubiquitin‐like PTM, important for activating Cullin‐RING type E3 ligases through NEDD8 conjugation.^11^, ^12^ Cullin‐RING E3 ligases are the largest family of E3 ligases that can generate signals for protein degradation.^13^ Parker et al. found that NEDD8 protein levels are increased during exercise and remain elevated during the recovery period. Interestingly, they showed that when NEDD8 is inhibited, protein ubiquitylation levels are not restored after cAMP stimulation (a signalling messenger often increased by exercise). These observations led them to hypothesise that exercise‐induced NEDDylation promotes protein ubiquitylation by activating Cullin‐RING E3 ligases which enhances the removal of damaged proteins.

To analyse global changes in exercise‐regulated protein ubiquitylation, Parker et al. used quantitative mass spectrometry. In order to detect these changes, they used diglycine (diGLY) antibody‐based immunoprecipitation to enrich ubiquitylated substrates at the peptide level. This approach uses monoclonal antibodies (K‐ε‐GG) which bind to diGLY remnants present on ubiquitylated peptides digested by trypsin.^14^ It is worth noting that the diGLY remnant is also a product of trypsin digested peptides modified by ubiquitin‐like modifications such as NEDDylation and ISGylation; however, the vast majority of diGLY enriched peptides are ubiquitylated.^15^ Once enriched, these modified peptides were then eluted and labelled using the isobaric chemical tandem mass tag (TMT) to enable precise quantification following mass spectrometry. This ubiquitin enrichment technique not only enhances the detection ubiquitylated peptides but also facilitates the identification of specific amino acid sites modified by ubiquitin.^16^ This technique has been used to identify ubiquitylated peptides and sites in several different cells^15^, ^17^ and a few rodent tissues.^18^ Within rodent skeletal muscle, employing diGLY antibody‐based immunoprecipitation has advanced our understanding of molecular signalling during atrophy.^19^, ^20^, ^21^ Parker et al. are the first to implement this technique in human skeletal muscle in the context of exercise.

The study by Parker et al. has provided a great platform for future research to employ recently developed techniques within the context of exercise to increase the depth of ubiquitylation profiling. Revealing ubiquitylation profiling in more depth will help towards understanding the role of regulatory proteins, for example the targets of exercise‐regulated E3 ligases, bridging existing knowledge in exercise‐induced PTMs and molecular signalling. This information will aid the discovery of exercise biomarkers and is useful for developing therapeutics to recapitulate certain benefits of exercise. With the continuous development of mass spectrometry‐based techniques, there is an opportunity to utilise highly sensitive methods to identify more ubiquitylated proteins and sites following exercise.

Udeshi et al. has developed an approach named UbiFast to improve the detection of ubiquitylated proteins by mass spectrometry.^22^ This approach uses diGLY antibody‐bound TMT labelling to ensure diGLY remnants are not labelled. TMT reagents are amine‐reactive and so bind to the amine present on diGLY remnants,^16^ which hinders the detection of diGLY‐modified peptides during mass spectrometry.^22^ When comparing this approach to the conventional labelling method employed by Parker et al., UbiFast detects a greater level of both total ubiquitylated peptides and their relative abundance.^22^ Importantly, UbiFast identified ~10,000 ubiquitylation sites from only 500 μg of peptides per sample derived from human tumour tissue.^22^ This demonstrates the high sensitivity of UbiFast for protein ubiquitylation profiling, which is beneficial when analysing small amounts of primary tissue samples such as human skeletal muscle.

To enhance ubiquitylation profiling, one could also consider directly enriching ubiquitin‐conjugated substrates at the protein level. Affinity enrichment of ubiquitylated proteins has been widely used prior to mass spectrometry by taking advantage of ubiquitin binding domains, such as Tandem ubiquitin‐binding entity (TUBE). TUBE contains a tetra‐ubiquitin binding domain structure that has a high affinity for all linkage types of polyubiquitin chains.^23^ However, TUBE‐based affinity enrichment of ubiquitylated proteins has limitations for use in mass spectrometry because TUBE itself will be digested during proteolysis. This creates excessive noise signals (peptides from TUBE) that mask the detection of ubiquitylated peptides. To overcome this problem, a trypsin‐resistant TUBE (TR‐TUBE) has been developed.^24^ TR‐TUBE was applied in cell to capture ubiquitylated proteins before diGLY antibody‐based immunoprecipitation prior to mass spectrometry. This dual enrichment approach improved the ratio of ubiquitylated peptides to total peptides detected compared to when only enriched at the peptide level, without reducing the total number of ubiquitylated peptides.^24^ Despite this, it is likely that the capture of mono and multi‐monoubiquitylated proteins was lower due to the selective binding of TR‐TUBE towards polyubiquitylated chains.^23^ This selective approach introduces bias towards profiling mainly polyubiquitylated proteins. To address this issue, one could employ MultiDsk instead of TUBE which has shown to effectively enrich both polyubiquitylated and monoubiquitylated proteins.^25^ Nevertheless, these findings provide clear evidence that this dual enrichment approach improved the purification of ubiquitylated peptides by reducing (non‐ubiquitylated) peptide background. Due to the complex protein biochemistry in skeletal muscle, it is important to remove non‐ubiquitylated proteins and peptides. Therefore, employing TR‐TUBE prior to diGLY antibody‐based immunoprecipitation may enhance the sensitivity of ubiquitylation profiling in skeletal muscle. We recommend applying this dual‐enrichment process with the UbiFast method (Figure 1).

**FIGURE 1:**
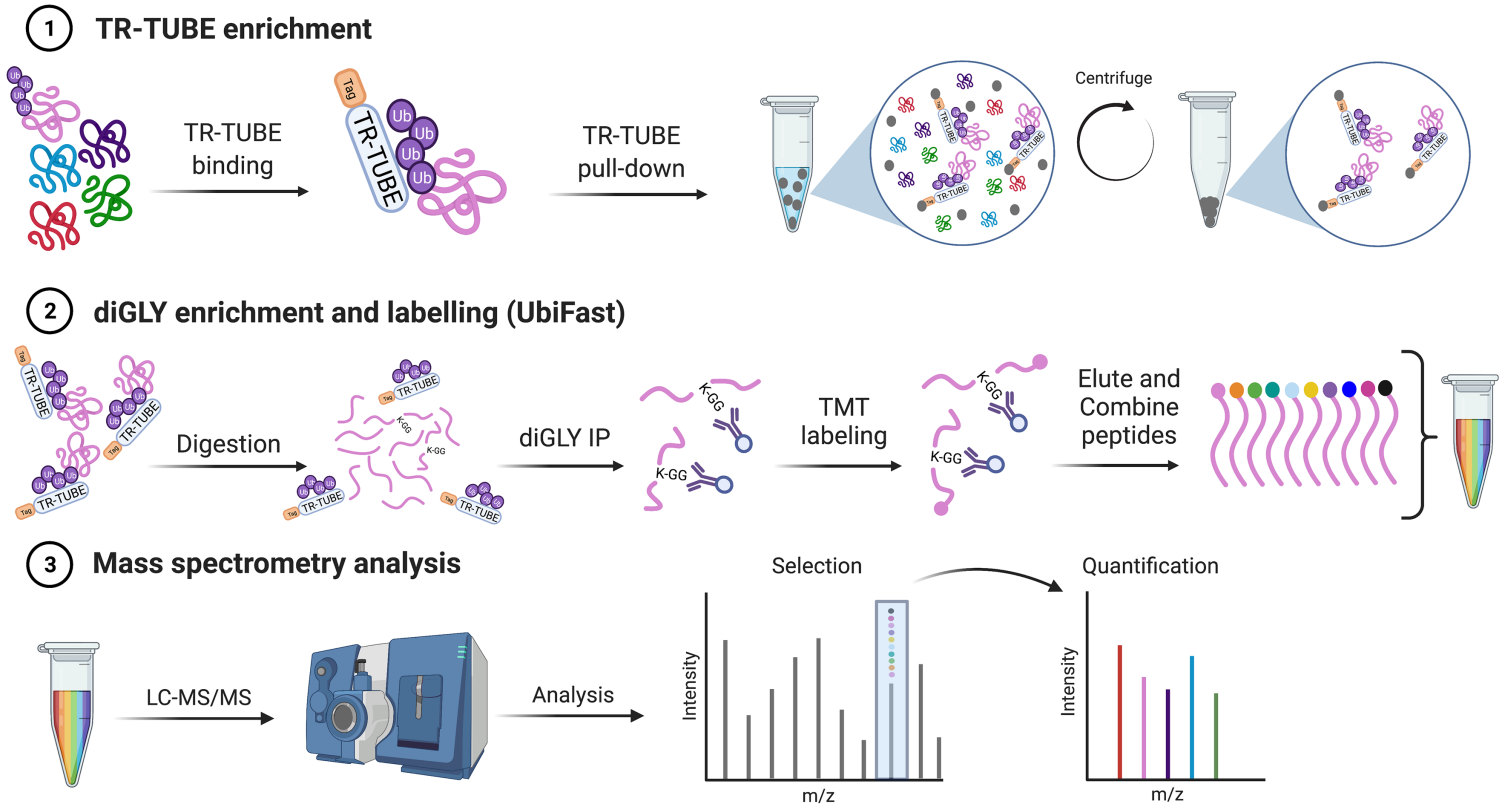
Schematic of proposed ubiquitin enrichment workflow for analysing exercise‐regulated ubiquitylation in skeletal muscle. Dual enrichment process whereby ubiquitylated substrates are first enriched at the protein level using TR‐TUBE pull‐down (1) and then enriched at the peptide level using diGLY antibody‐based immunoprecipitation (2). To enable quantification of the ubiquitylated peptides, isobaric TMT labelling is performed while peptides are bound to the diGLY antibody. Labelled peptides are analysed by LC–MS/MS (3). Created with BioRender.com

Another approach that could be taken to enhance our understanding of exercise‐induced ubiquitylation is to investigate specific ubiquitin chain type signalling. As mentioned previously, different ubiquitin chain types can signal for different biological processes.^26^ Therefore, identifying which chain type is present on ubiquitylated proteins is important for understanding the functional role of ubiquitylation. There are different TUBE‐like enrichment tools that are capable of purifying specific ubiquitin chain types.^27^ As such, these tools would be useful for investigating chain‐specific substrates following exercise. In fact, the importance of chain specificity has been highlighted in the Parker et al.’s study showing that K27‐linked ubiquitin chains increased during high‐intense exercise.^10^ More recently, advances in ubiquitin chain type screening methods have revealed that K33‐linked ubiquitin chains are highly enriched in skeletal muscle relative to other tissues.^28^ These findings suggest that atypical ubiquitin chains such as K27 and K33‐linked ubiquitin chains may have a unique role to play in skeletal muscle. Investigating different ubiquitin chain types in more detail will help discover new regulatory aspects of exercise‐induced ubiquitylation. Notably, not all chain types currently have an effective tool for enrichment, meaning only a select few can be analysed in this manner.^27^ Therefore, this is an area that requires further development.

Alongside sample preparation, the method to which data is acquired from the mass spectrometer can also alter the sensitivity of detecting ubiquitylated peptides. There are different quantitative methods that can be employed in mass spectrometry, discussed in more detail in other review papers.^29^, ^30^ Typically, quantitative mass spectrometry is performed using data‐dependent acquisition (DDA) which detects peptides through intensity‐based selection. Parker et al. employed this technique by comparing the relative ion intensities between TMT‐labelled peptides. Despite the high spectral quality, this selective approach has been known to cause stochastic peptide selection and chemical noise production.^31^, ^32^ As a result, this form of data acquisition can lead to missing values and a reduced dynamic detection range. Alternatively, data‐independent acquisition (DIA) identifies peptides simultaneously within a fixed mass‐to‐charge range. This method has proven to increase signal‐to‐noise ratio and peptide selectivity resulting in more sensitive and accurate quantification.^31^ Recently, The DIA approach has been able to identify up to 70,000 ubiquitylated peptides from as little as few hundred µg to 1 mg of protein obtained from human non‐muscle cell lines.^33^, ^34^ However, obtaining such a comprehensive ubiquitin profile requires a project‐specific peptide spectral library or leveraging a neural network prediction for identification and subsequent quantification. Moreover, TMT labelling is not suitable for DIA‐based quantification and so this method would not be compatible with the UbiFast approach. Nonetheless, with sufficient sample availability and the available skills for analysing the complex MS/MS spectra, this highly sensitive DIA‐based approach could be considered to increase the detection of ubiquitylated peptides in exercised skeletal muscle.

In conclusion, the study by Parker et al. has provided advanced insights into exercise‐regulated ubiquitin signalling, revealing its dynamic nature and the potential regulatory role of NEDDylation in skeletal muscle. In addition to validating the newly identified ubiquitin substrates, future work can implement more sensitive mass spectrometry‐based workflows to improve the detection of ubiquitylated sites, peptides and specific chain types. This will advance our understanding of the exercise‐regulated ubiquitin signalling network, revealing molecular mechanisms that control structural and metabolic skeletal muscle functioning.

## CONFLICT OF INTEREST

The authors report no conflicts of interest.

## AUTHOR CONTRIBUTIONS

S. Lord: conceptualisation, visualisation, writing original draft, writing review and editing. Y‐C. Lai: conceptualisation, writing review and editing. Both the authors contributed to the article and approved the submitted version.
